# Endophytic fungi: a tool for plant growth promotion and sustainable agriculture

**DOI:** 10.1080/21501203.2021.1945699

**Published:** 2021-06-29

**Authors:** Noemi Carla Baron, Everlon Cid Rigobelo

**Affiliations:** Agricultural and Livestock Microbiology Post Graduation Program, Department of Plant Production Sciences, School of Agricultural and Veterinarian Sciences, São Paulo State University (UNESP), Access Way Prof. Paulo Donato Castellane, São Paulo, Brazil

**Keywords:** Endophytic fungi, Biocontrol, Biofertilization, Plant growth, Sustainable agriculture

## Abstract

Endophytic fungi are found in most, if not all, plant species on the planet. They colonise inner plant tissues without causing symptoms of disease, thus providing benefits to the host plant while also benefiting from this interaction. The global concern for the development of more sustainable agriculture has increased in recent years, and research has been performed to decipher ecology and explore the potential of endophytic interactions in plant growth. To date, many studies point to the positive aspects of endophytic colonisation, and in this review, such research is summarised based on the direct (acquisition of nutrients and phytohormone production) and indirect (induced resistance, production of antibiotics and secondary metabolites, production of siderophores and protection for abiotic and biotic stresses) benefits of endophytic colonisation. An in-depth discussion of the mechanisms is also presented.

## 1. INTRODUCTION

The association between plants and fungi is extremely common. Fossil records indicate the existence of this union with endophytes and mycorrhizas have existed for more than 400 million years (Krings et al. 2007; Chadha et al. 2015) starting when plants colonised the soil, thus indicating the importance of this group in the evolution of this process (Rodriguez et al. 2009; Rai et al. 2014; Anjum et al. 2019). The positive aspects of this interaction have always been noted and discussed, but in-depth studies evaluating the real benefit provided by these fungi have only been performed recently (Busby et al. 2016; Card et al. 2016; Vega 2018; Quesada-Moraga 2020).

Several characteristics of the fungal endophytic interaction still need to be fully elucidated, but fortunately, science is advancing in the search for this understanding (Aly et al. 2011; Chadha et al. 2015; Khan et al. 2015). Awareness about the need for more sustainable agriculture is the main incentive for the recent scientific research, and improving sustainable agriculture should help to protect and reduce the negative impacts on the environment in the future (Jaber and Enkerli 2017; Baron et al. 2020).

The uncontrolled and inadequate application of pesticides and fertilisers harms the environment and human health. Residues from these processes can be found in foods, such as vegetables, fruits, cereals, and grains, and even byproducts such as juices and wines, depending on the practices adopted for their production (Zikankuba et al. 2019). In their review, Sabarwal et al. (2018) described several studies relating the occurrence of various human health disorders in children, adults and the elderly to exposure to pesticides, including Hodgkin’s disease, lymphoma, Parkinson’s disease, endocrine disorders, respiratory and reproductive problems in addition to cancer. In addition, nontarget organisms are constantly affected, such as in the aquatic environment, including zooplankton, crustaceans and fish, or terrestrial environments, including natural pollinators, livestock, birds and beneficial microorganisms present in the soil (Van Lexmond et al. 2015). In addition, the excessive use of fertilisers leads to the accumulation of heavy metals, the eutrophication of rivers and lakes, the acidification of soils, the contamination of aquifers and water reservoirs, and the generation of gases associated with the greenhouse effect (Savci 2012; Kulkarni and Goswami 2019).

Knowledge about the symbiotic relationship between plants and soil microbiota and the synergistic mode of action representing a positive interaction has been fundamental in the search for alternative processes that could be used to reduce or even replace the application of pesticides and fertilisers to develop more sustainable agriculture (Carneiro et al. 2015; Ahmad et al. 2018). In this context, studies have been conducted beyond the potential use of microorganisms as classical biological control agents (BCAs) through inundative inoculation in crops. For example, the fungal agents *Metarhizium anisopliae* and *Beauveria bassiana* are the best characterised and most commonly used entomopathogenic fungi in biological control programmes for the control of arthropods that can act as pests or disease vectors (Baron et al. 2019; Quesada-Moraga 2020). However, several reports have shown that these fungi and other known entomopathogens can protect plants by direct interactions between the fungi and the plants. In this case, fungi are able to act as antagonists of plant pathogens through the use of a diverse range of mechanisms, such as the production of metabolites (antibiotics, volatile compounds and enzymes), engagement in competition (for space, carbon sources, nitrogen and minerals) and parasitism, induction of systemic resistance by the plant and increases in plant growth, resulting in the reduction of the activity of the pathogens (Vega et al. 2009; Vidal and Jaber 2015; Vega 2018; Lr 2018; Quesada-Moraga 2020).

From this perspective, studies are focusing on characterising endophytic fungal community of several plant species, especially those of agronomic interest. Studies with endophytes not only reveal very interesting aspects about the ecology and the way that these microorganisms interact with plants but also help to understand the benefits that can result from this interaction and the factors that should be explored for the development of sustainable processes among human practices, especially agriculture. The characterisation of endophytic fungi expands the possibility of their use not only as biocontrol agents but also as biostimulants and biofertilizers. In this review, aspects of the endophytic way of life and how scientific research is evolving to elucidate the potential use of endophytic organisms for crop development and commercialisation as bioproducts for agriculture will be discussed.

## 2. Fungal endophytes: definition, classification, biodiversity and distribution

The meaning of the term endophytic has been well discussed and different definitions have been proposed. In general, it used to be applied to any organism that lives inside (é*ndon*) of a plant (*phyton*), as originally postulated by De Bary (1886). The term endophytic is currently related to microorganisms that inhabit internal plant tissues, including bacteria, fungi, viruses, protozoa and even microalgae and do not cause disease symptoms in their host (Hyde and Soytong 2008; Rodriguez et al. 2009; Hardoim et al. 2015). Scientists are in a deep discussion about the use of the term endophyte nowadays, because for certain groups the term should refer to habitat only, and not function (Hardoim et al. 2015), while others even suggest the adoption of the term “mutualistic endophytes”, considering that endophytic microorganisms are those that provide some benefit to their host, with the term “endophytic” excluded for latent or dormant pathogens or saprophytes (Card et al. 2016). A more recent definition proposed by Le Cocq et al. (2017) concludes that endophytes are microbes which inhabit internal plant tissues for at least part of their life cycles and cause no harm to the host plant under any circumstance, meaning that those microbes currently considered as endophytes but which present harmful effect to a plant host at any moment should have their designation changed.

The endophytic interaction is defined as balanced antagonism (Schulz et al. 2015) because the recognition of the plant as a host requires the activation of virulence mechanisms for colonisation and the triggering of host defences by these events. While an equilibrium exists in this interaction, the fungus survives of nutrients from the host plant and, in exchange, provides benefits, including tolerance to biotic and abiotic stresses (Bamisile et al. 2018).

Endophytic fungi are divided into clavicipitaceous (usually associated with grasses) and nonclavicipitaceous (not found in grasses) (Hyde and Soytong 2008). Rodriguez et al. (2009) classified clavicipitaceous and nonclavicipitaceous fungi into 4 classes (Table 1): class 1 contains all the clavicipitaceous fungi that are specific colonisers of grasses, and they can be found in the aerial part and/or roots of their hosts and are transmitted horizontally and vertically; and classes 2, 3 and 4 include noncclavicipitaceous fungi. Class 2 consists of endophytes capable of colonising the aerial part and roots, and they are transmitted horizontally and vertically. Class 3 consists of endophytes commonly associated with the leaves of tropical tree species, a very diverse group that has only horizontal transmission. Finally, class 4 includes so-called dark septate endophytes, fungi that have melanin in their septa and occur exclusively in the roots of their hosts and present only horizontal transmission. Recently, Lugtenberg et al. (2016) suggested the inclusion of an additional class for endophytic entomopathogenic fungi because they are able to grow as symptomless endophytes of several plant species and present the unique ability to infect and colonise insects.

**Table 1.** Summary of characteristics of endophytic fungi classes according to Rodriguez et al. (2009)

Class of endophytes	Main fungal genera	Common host/ colonised tissues	Transmission	Reference
Class 1	Epichloë, Metarhizium, Claviceps and others	Grasses /shoot and roots	Horizontally and vertically	Faeth and Saari (2012) Behie and Bidochka (2014)
Class 2	Phylum Ascomycota Penicillium, Aspergillus, Fusarium, Colletotrichum, Trichoderma, Beauveria, Purpureocillium, and others Phylum Basidiomycota Xylaria spp.	Great host range/ roots, stem and leaves	Horizontally and vertically	Rodriguez et al. (2009) Hiruma et al. (2018) Waqas et al. (2012) Khan et al. (2008) Dash et al. (2018) Lopez and Sword (2015)
Class 3	Sobreposition with Class 2 endophytes in many cases. It depends on the host, local of infection in the plant and mode of transmission	Mainly tropical trees/ leaves	Horizontally only	Rodriguez et al. (2009)
Class 4	Curvularia, Alternaria, Phialocephala, Deschlera, Ophiosphaerella, Cladosporium, and others	Great host range/ Roots	Horizontally only	Rodriguez et al. (2009) Hamayun et al. (2010) Spagnoletti et al. (2017)

Bamisile et al. (2018) gathered information from several studies and proposed that endophytic fungi can be classified according their ecology, diversity and function. They can be classified as **sexual or asexual** according to the mode of reproduction by sexual or asexual spores. Additionally, they are **horizontally or vertically transmitted** based on their mode of transmission. Horizontal transmission occurs when vegetative propagules or spores are produced by the endophyte and spread to the plant population through the air or via some vector, while vertical transmission consists of the transference of the fungi to the plant progeny via seeds (Gimenez et al. 2007; Aly et al. 2011; Lugtenberg et al. 2016). In relation to the expression of infection, fungal endophytes can be classified as **symptomatic or asymptomatic** and as **root or foliar endophytes** depending on the part of the plant that is colonised. Finally, they can be termed **biotrophic or necrotrophic** according their mode of nutrition, with biotrophic endophytic fungi obtaining nutrients from living tissues and necrotrophic fungi promoting necrosis to grow from dead tissues (Kemen and Jones 2012).

Studies involving clavicipitaceous fungi (Class 1) are more widespread, and much is known about their interactions with grasses, especially for the *Epichlöe* genus (KD and Soytong 2008; Rodriguez et al. 2009; Card et al. 2014; Zhang et al. 2015; Lugtenberg et al. 2016; Chitnis et al. 2020). The main benefit to plants by these fungi is the production of secondary metabolites, mainly alkaloids, that accumulate in plant tissues and present bioactivity against many vertebrates, invertebrates and other pathogens (e.g. fungi) and can also confer tolerance to abiotic stresses (Card et al. 2016). Currently, *Epichloë* species are commercialised worldwide for the cultivation of these grasses (Johnson et al. 2013; Finch et al. 2016). Among nonclavicipitaceous fungi, research on the knowledge of their endophytic relationship with plants and the analysis of benefits that can be explored in the agricultural context are more widespread among representatives of class 2, which includes several species taxonomically belonging to the subkingdom Dikarya, which includes the phyla Ascomycota and Basidiomycota (Rodriguez et al. 2009).

In relation to their occurrence and biodiversity, many aspects remain unknown. Endophytic fungi have already been recovered from a wide range of habitats, including artic environments, hot deserts, and mangrove, temperate and tropical forests (Arnold and Lutzoni 2007; Arnold 2008). As reviewed by Chadha et al. (2015) and Lugtenberg et al. (2016), the characterisation of the diversity and the distribution of fungal endophytes across large geographical areas is still in the beginning, and only some general aspects can be affirmed, such as that the diversity of endophytic fungi is higher in the tropics than in higher latitudes. Additionally, a higher number of endophytic species are found in tropical environments and belong to a small number of classes.

## 3. Specificities of endophytic colonisation and the characterisation of endophytic biodiversity

The diversity of endophytic fungi associated with plants can greatly vary according to environmental conditions (Vega et al. 2010), i.e. even for plants of the same species, the assemblage of fungal endophytes inside their tissues can vary if the physiological state of each individual is different (Aly et al. 2011). Moreover, the age of the plant can also influence the fungal endophytic community profile (Sieber, 2007).

Some fungal endophytes are able to colonise a wide range of plant species, while others are more specific and occur only inside a restricted number of plants. Additionally, specificity can also be present in relation to the portion of the plant that is colonised (Aly et al. 2011; Bamisile et al. 2018). Apparently, vertically transmitted fungi seem to present plant associations with a more mutualistic profile than horizontally transmitted fungi, which are more likely antagonists (Aly et al. 2011).

The genetic communication between the endophyte and the host plant for the establishment of the interaction is a complex and poorly understood process that involves the selective expression of fungal genes responsible for the production of enzymes and secondary metabolites that aid in colonisation (Bayle et al., 2006; Yan et al. 2019). The approximation of the germinative tube of the endophyte to the root causes the loss of apical dominance of the root and the formation of a hyphal penetration apparatus (aspersorium), which enters the root cortex with hyphae of infection, thereby starting the colonisation process (Khan et al. 2015). These events promote a balanced activation of plant defence genes, and when the fungus reaches the inner cortex, the hyphae penetrate the plant cell wall and continue the colonisation of adjacent tissues (endoderm, pericycle, xylem, phloem) of the roots and of the soil (Khan et al. 2015; Yan et al. 2019).

Particular aspects demonstrate the closeness in the relationship between the plant and their fungal endophytes, including the lack of defence reactions against them, the ability of some endophytes to produce metabolites of the plant host (Germaine et al. 2004; Kusari et al. 2012) and even the simultaneous occurrence of both reproductive processes (fungi and plant host) in the case of vertical transmission via seeds (Aly et al. 2011).

In relation to the analysis of the fungal endophytic community of plants, it is certainly a big challenge that has been progressively overcome by science. The presence of these microorganisms within plant tissues is difficult to be visualised, the hyphae are rarely observed and distinctive characteristics are scarce (Rashmi et al. 2019). The endophytic community has been traditionally assessed through isolation from surface-sterilised plant tissues, aiming for the recovery of fungal strains present only in the inner of the plant. Therefore, conventional culture media are used, including modifications when necessary, such as the addition of a higher proportion (usually double) of water in the medium aiming to avoid an osmotic chock and favour the access of exploratory hyphae. Further, the addition of plant extracts in the culture media can be adopted (Murphy et al. 2018).

The characterisation of the endophytic fungi diversity in a host plant by cultivation-dependent methods is considered limited and can be influenced by several biotic and abiotic factors (Ribeiro et al. 2018; Rashmi et al. 2019; Chen et al. 2020). Therefore, the introduction of the use of molecular tools to identify the endophytic community of different plant species has excelled in scientific surveys. The investigation of microbiomes has been performed through mass DNA sequencing from plant material, without needing cultivation and it allows the identification of a great number of uncharacterised endophytic taxa (Brader et al. 2017; Deyett and Rolshausen 2020).

## 4. From biocontrol to biofertilization and stress tolerance: how can endophytic fungi help?

Several regions in the world have experienced a decrease in water availability and an increase in soil salinisation and desertification in addition to other problems related to the excessive soil use, deforestation and inappropriate irrigation practices (Chadha et al. 2015; Lugtenberg et al. 2016). A method of resolving these issues is the development of new plant varieties by breeding wild plants. However, the genetic mechanisms involved in stress tolerance are poorly known, and an essential aspect is not considered in this process: the symbiotic association of plants and microorganisms (Chadha et al. 2015). The fungal endophyte community that exists in wild plants can be severely modified, and many representatives can be lost during domestication; therefore, fungi are harmed by losing their safe niche and plants are deprived of a partnership that could improve their ability to overcome environmental challenges (Lugtenberg et al. 2016). For a deeper discussion about the reasons why endophytes can be lost during plant breeding, see Lugtenberg et al. (2016).

For endophytes, the inner part of the plant is a protected niche that contains the necessary nutrients for fungal survival and growth in addition to presenting low competition with other microorganisms. Therefore, in exchange for this safe place, fungi improve plant fitness by several mechanisms (Khan et al. 2015; Lugtenberg et al. 2016; Chitnis et al. 2020). The benefits of plant colonisation by endophytic fungi can occur directly and/or indirectly, and the differentiation among them is complex (Berg 2009). Among the direct mechanisms of growth promotion, the most important are the acquisition of nutrients and the production of phytohormones, while tolerance to biotic and abiotic stresses, including combat against pathogens, is considered an indirect aspect in the promotion of growth (Hardoim et al. 2015; Souza and dos Santos 2017).

Considering the difficulties of plant breeding for many crops and the fact that their use is still restricted in many countries because the effects of genetic modification on human, animal and environmental health are not totally understood (Chadha et al. 2015), the development of natural alternatives certainly has great promise for achieving more sustainable agriculture. The use of endophytic fungi is just starting, and in the following sections, the direct and indirect mechanisms by which these microorganisms can aid in plant health will be detailed, including a perspective of their use in agricultural processes in the near future.

**Figure 1.**
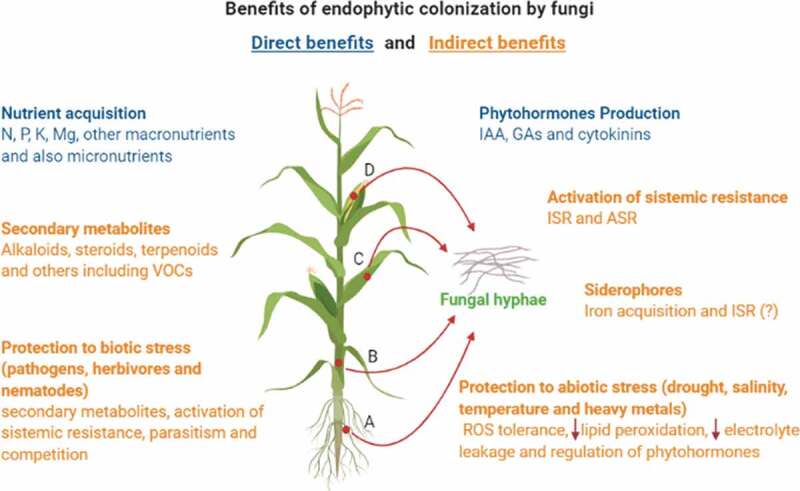
Benefits provided to plants by endophytic colonisation with fungi. Maize is indicated as an example once endophytic interaction may benefit different crops. Endophytic colonisation can occur in the tissues of one or more parts of the host plant, including roots (A), stem (B), leaves (C), reproductive systems, and fruits (D). From the inner of plant tissues, fungi can contribute directly or indirectly to different ways to plant fitness and growth promotion. Direct benefits from this interaction are indicated in the blue colour text while indirect benefits are indicated in the orange colour text. Figure was created with BioRender.com.

The mechanisms described below can be summarised in Figure 1.

The **direct benefits** of interaction with endophytic fungi include the increase in acquisition of nutrients and in the amount of phytohormones in the plant, which is directly related to the increase in biomass production, expansion of root system development, plant height, weight reproduction and yield. Because of these benefits, they can be referred to as biofertilizers (Bamisile et al. 2018).

### a) Acquisition of nutrients

– Endophytic fungi are able to improve the uptake of macronutrients, such as phosphorus, nitrogen, potassium and magnesium, or micronutrients, such as zinc, iron, and copper, from the soil and organic matter and increase the supply of these nutrients to the plant host (Rana et al. 2020).

Behie et al. (2012) provided the first report of the endophytic colonisation of bean plants (*Phaseolus vulgaris*) and switchgrass (*Panicum virgatum*) by the entomopathogenic fungus *Metarhizium robertsii* and the way in which it was able to transfer nitrogen (N) from *Galleria mellonella* larvae, which are infected and killed by it. Since then, new studies have been carried out, such as by Behie and Bidochka (2014), who evaluated the endophytic transfer of N from insects by seven fungal species, namely, *M. robertsii, M. guizhouense, M. brunneum, M. flavoviridae, M. acridum, B. bassiana and Akanthomyces (=Lecanicillium) lecanii*, to four cultures consisting of two dicots, *Glicyne max* (soybean) and *P. vulgaris* (common bean), and two monocots, *Triticum aestivum* (wheat) and *P. virgatum* (switchgrass). As a result, the authors found that five species of *Metarhizium* and *B. bassiana* can killing insect larvae and endophytically colonising plants and carry out the transfer of N from insects to these plants.

*Serendipita* (=*Piriformospora*) *indica* is a well-characterised endophyte that has nutrient transportation abilities described in the literature, including the delivery of phosphates to host plants (Card et al. 2016). Despite the lack of specific studies on phosphorus (P) transference by endophytic fungi, many reports indicate the improvement in P acquisition by fungal inoculation and presume the occurrence of this process by an endophytic interaction. Ortega-Garcia et al. (2015) demonstrated that the inoculation of *Trichoderma asperellum* significantly reduced the use of phosphorus fertilisation in onion (*Allium cepa*). Similarly, Baron et al. (2018) performed a field study and inoculated maize (*Zea mays*) with *Aspergillus sydowii*, and the plants that interacted with the fungus accumulated significantly higher amounts of P in their tissues even when receiving lower fertilisation doses.

In relation to the mechanisms of nutrient transportation, exact metabolic pathways and molecules involved in many processes have not yet been well described. As an example, Hiruma et al., 2018 compared in their review the transport of P among plants colonised by AMF (Arbuscular Micorrhyzal Fungi) and by Brassicaceae plants (which evolutionarily lost their association with AMF) colonised by *Colletotrichum tofieldiae*. In AMF-colonised plants, P transporters are overexpressed in plants, and they accumulate on the biotrophic surface (arbuscules), while in *Arabidopsis thaliana* colonised with *C. tofieldiae*, several genes related to P transport present an increase in their expression (e.g. *PHT1;2* and *PHT1;3*); however, it remains unclear whether transporters accumulate in the biotrophic surface and whether they are necessary in the growth promotion process mediated by endophytic colonisation.

### b) Production of phytohormones

– Endophytic fungi are able to produce auxins, gibberellins (GAs) and cytokinins. The potential of phytohormone production by endophytic fungi is underexplored, especially for gibberellins, even though these molecules are as important as chemical signalling and messengers for plant growth in different environmental conditions (Khan et al. 2015).

The main auxin produced by fungi is indole-3-acetic acid (IAA). Auxins are the main regulators of plant growth and present several positive effects on shoot and root development, such as the responses of tropism, division and cell elongation, differentiation of vascular tissue and initiation of the root formation process (Jaroszuk-Ściseł et al. 2014). Waqas et al. (2012) reported IAA and GA production by the endophytic fungi *Phoma glomerata* and *Penicillium* sp. The production of IAA and GAs was also described for the endophytic fungus isolated from cucumber plants *Paecilomyces formosus* (=*P. maximus*) strain LHL10 (Khan et al. 2012). The main precursor of IAA biosynthesis by endophytic fungi is L-tryptophan, but the metabolic pathway used for IAA production has not been described, indicating the importance of further investigations on this theme (Numponsak et al. 2018).

Gibberellins are essential in several plant responses, including seed germination, stem elongation, sexual expression, flourishing, fruit formation and senescence (Bömke and Tudzynski 2009). The production of gibberellins by endophytic fungi is described as occurring from acetyl-CoA by the mevalonic acid (MVA) pathway, and the main final products are GA_1_ and GA_3_, which are produced from GA_4_, GA_5_ and GA_7_ (Bömke et al. 2008; Khan et al. 2008, 2015).

Khan et al. (2008) detected gibberellin production by *Penicillium citrinum* IR-3-3. The fungus was isolated from dune plants and screened among 15 isolates. *P. citrinum* IR-3-3 was able to promote growth, thereby improving the length of seedlings of the Waito-c rice dwarf mutant (which is deficient for gibberellin production) and in the common sandy plant *Atriplex gmelinii*. The production of the bioactive gibberellins GA_1_, GA_3_, GA_4_ and GA_7_ was detected in fungal extracts at a higher level than in extracts of the known GA producer *Gibberella fujikuroi* used as positive control in the study, which did not promote growth as *P. citrinum* IR-3-3. Gibberellin production was also described for *Aspergillus fumigatus* (strain HK-5-2) (Hamayun et al. 2009a) and *Cladosporium sphaerospermum* (strain DK-1-1) (Hamayun et al. 2009b), indicating their ability to improve the plant growth of soybean plants. In addition, Hamayun et al. (2010) reported gibberelin production by an endophytic isolate of *Cladosporium* sp. MH-6 and the positive effect on the growth of cucumber plants by applying culture filtrates of the fungus.

Recent studies, such as Bader et al. (2020), describe not only a single ability, such as the increase in nutrient uptake or phytohormone production, but also two or even more abilities presented by the same strains. The authors isolated *Trichoderma* strains from Argentine Pampas soil and selected four strains that presented high IAA production and P solubilisation capacity. The strains were inoculated in tomato seeds, and 45 days after germination, the plants that received the fungi presented a significant increase in plant height, fresh and dry matter of shoots and roots and chlorophyll content in the leaves in addition to a higher surface area.

Baron et al. (2020) also demonstrated in their study the ability of *Purpureocillium lilacinum, Purpureocillium lavendulum* and *Metarhizium marquandii* to produce IAA and solubilise P from fluorapatite. The selected strains were tested in soybean, bean and maize plants and were able to promote growth-improving parameters such as dry matter (shoot and roots) and the availability of important nutrients such as P and N. The authors attribute the growth promotion to endophytic colonisation of the plants.

Among the **indirect benefits** of interaction with endophytic fungi:

### a) Activation of systemic resistance

– Endophytic fungi can aid plants in improving their self-defence system, thus promoting the activation of induced systemic resistance (ISR) pathways, which may overlap with that of acquired systemic resistance (ASR) because both systems can improve plant growth (Berg 2009; Busby et al. 2016) and protect against pests and pathogens (Chadha et al. 2015).

The activation of systemic resistance initiates with the recognition of pathogen-related molecules, named pathogen-associated molecular patterns (PAMPs) (Poveda et al. 2020). For microorganisms, the term MAMPs is used to refer to microbe-associated molecular patterns, which are recognised by plant receptors and induce the so-called MAMP-triggered immunity (MTI). Examples of MAMPs that induce MTI are chitin and β-glucan of the fungal cell wall, secreted enzymes (xylanases, glucanases and chitinases) and their products (Latz et al. 2018; Yan et al. 2019). In addition to MAMPs, effector molecules (e.g. secondary metabolites) produced by microorganisms can induce resistance, activating effector-triggered immunity (ETI) (Yan et al. 2019; Poveda et al. 2020). The MTI response is similar to that of endophytic and pathogenic microorganisms, but it has been noticed that among endophytic fungi, a modification in MAMPs can occur, so it is not recognised by the plant (Yan et al. 2019). For example, the MAMP β-glucan of the cell wall of *S*. (=*P*.) *indica* can be modified by the expression of the *FGB1* gene, which encodes a fungal-specific β-glucan-binding lectin. This modification alters the composition and properties of the endophytic cell wall and is enough to suppress MTI in different plant hosts (Wawra et al. 2016). For ETI, it is suggested that beneficial microorganisms are able to overcome this barrier, thereby facilitating the process of colonisation (Yan et al. 2019).

The activation of ISR and ASR pathways by MAMPs and effectors leads to a complex response that is not largely known but involves the flux of ions, the phosphorylation and dephosphorylation of proteins, the production of signalling molecules (such as ethylene and salicylic acid) and reactive oxygen species (ROS) and the selective expression of genes that are conducive to defence responses, such as the thickening of the plant cell wall, the production of pathogen-related (PR) proteins and phytoalexins and even cell death (Chadha et al. 2015). In this context, endophytic colonisation presents a priming effect, preparing the plant for further infections by pathogenic microorganisms, herbivores or nematodes (Latz et al. 2018; Poveda et al. 2020).

The balanced interaction between fungal endophytes and their plant hosts occurs due to the lack of pathogenic properties. A good example is the comparison of Brassicaceae’s endophytic strain *Colletotrichum tofieldiae* and the pathogenic *Colletotrichum incanum* in *Arabidopsis thaliana*. Evolution has negatively selected genes of effector proteins in the endophytic strain, which are directly involved in the pathogenic action at the moment of plant colonisation, and the same did not occur with the pathogenic strain *C. incanum*. On the other hand, Brassicaceae plants also present fewer receptors for these proteins. Therefore, the maintenance of these genes in pathogenic species may represent a potential strategy for host attack, while species with the tendency to develop beneficial interactions with plants reduced the repertoire of these genes in their genome (Hiruma et al. 2018).

### b) Production of antibiotics and secondary metabolites

– In addition to stimulating the production of defence molecules by the plant itself, endophytic fungi are a large reservoir of molecules that act in favour of their host. They are excellent producers of compounds with activity against pathogens and herbivores, including alkaloids, steroids, terpenoids, peptides, polyketones, flavonoids, quinols, phenols, chlorinated compounds and volatile organic compounds (VOCs) (Card et al. 2016; Lugtenberg et al. 2016; Latz et al. 2018; Kaddes et al. 2019). Moreover, studies report the production of compounds with antiviral, antibacterial, antifungal and insect action (Card et al. 2016; Latz et al. 2018).

The best-known example of secondary metabolites produced by endophytic fungi is alkaloid production by *Epichloë* species in different species of grasses. Alkaloids accumulate in plants and are toxic to several insect pests and even vertebrates (Faeth 2002; Gimenez et al. 2007; Johnson et al. 2013; Lugtenberg et al. 2016). The production of nodulisporic acid by *Nodulisporium* sp. is also reported. This molecule is important for controlling insect herbivory because it activates glutamate in the chlorine channels of insect muscle and nerve cells. The activation of glutamate leads to the flow of chlorine ions through the channels, which results in flaccid paralysis (Demain 2000). An uncountable number of molecules are produced as secondary metabolites by endophytic fungi; however, specific pathways and substances have not been well characterised thus far. The review of Lugtenberg et al. (2016) is recommended for deeper knowledge of the chemical structures of some secondary metabolites produced by endophytic fungi.

Among the wide range of secondary metabolites produced by endophytic fungi, more than 300 of these molecules are VOCs (Lugtenberg et al. 2016; Kaddes et al. 2019). They consist of small molecules, presenting high vapour pressure, and they are easily diffusible through the cell membrane, in the atmosphere and in the soil, which makes them special agents of fungal communication with other organisms, including plants, in addition to presenting bioactivity against many pathogens (Kaddes et al. 2019). Strobel et al. (2001) introduced the concept of mycofumigation, a biocontrol technique to be used in the control of postharvest diseases of fruits and tubers. As reviewed by Kaddes et al. (2019), the genus *Muscodor* is the most explored in relation to the production of VOCs, presenting a wide range of these metabolites. This fungus has been used in the postharvest process and as a soil inoculant where it inhibits the growth of pathogenic fungi by VOC production. Moreover, the genus *Nodulisporium* is recognised by producing VOCs with antifungal activity and has been applied with the same purposes as *Muscodor*.

Secondary metabolites are produced either for signalling or defence or in the process of establishing their interaction with the host plant. In addition, they can influence the profile of secondary metabolites produced by host plants, which can, for example, directly influence the attack of a pathogen. Several chemical synthetic compounds used in agriculture are harmful to humans, animals and environmental health, and many of these molecules are prohibited; thus, similar products will likely no longer be commercialised in the future (Lugtenberg et al. 2016). Therefore, research and enterprises are attempting to exploit and transform biological products as an alternative for more sustainable agriculture.

### c) Production of siderophores

– Iron is an essential microelement for all living cells (Rana et al. 2020; Turbat et al. 2020). Siderophores are small molecules that present iron-chelatin properties and are produced by some microorganisms, including endophytic fungi, to bind ferric ions in the rhizosphere (Chowdappa et al. 2020; Sr et al. 2020).

Suebrasri et al. (2020) detected the production of siderophores by endophytic strains of *Trichoderma koningii* ST-KKU_1_, *Macrophomina phaseolina* SS_1_L_10_ and *M. phaseolina* SS_1_R_10_. In this study, the authors suggested that siderophore production by fungi was important in the growth promotion of sunchoke plants. The production of siderophores is also described for recombinant *Trichoderma harzianum* endophytic strains colonising beans (*P. vulgaris*) (Eslahi et al., 2020). The function of siderophores produced by fungal endophytes is still poorly known and characterised, and their relationship with the ISR is speculated (Card et al. 2016).

### d) Protection against biotic and abiotic stresses

– Environmental degradation by agricultural processes and global climate change expose plants to increasingly challenging conditions for their growth and maintenance. Moreover, the situation is even more difficult for crops because higher yields are increasingly being required. In this scenario, it is clear that some aid is necessary for good plant development, and endophytic fungi are a promising alternative for plant protection from biotic and abiotic stresses.

Endophytic fungi are able to combat abiotic stresses, including drought, high and low temperatures, salinity and toxic heavy metals (Aly et al. 2011; Khan et al. 2015). For biotic stress protection, fungal endophytes are responsible for the activation of ISR and ASR, which produce metabolites against pathogens; moreover, parasitism or competition can occur to avoid disease and herbivory (Chadha et al. 2015; Chitnis et al. 2020).

Abiotic stresses are responsible for negative impacts on plant morphology and physiology due to genetic regulation of cell pathways that cause several dysfunctions (Egamberdieva et al. 2017). Endophytic fungi help host plants adapt to stress conditions through diverse mechanisms. As reviewed by Khan et al. (2015) and Yan et al. (2019), during oxidative stress, plants increase the activity of antioxidant enzymes, mainly catalases and peroxidases, which leads to the production of ROS, resulting in membrane attack causing the peroxidation of membrane lipids. By some not yet defined mechanisms, endophytic fungi confer tolerance to ROS, reducing lipid peroxidation. Another important problem caused by abiotic stresses (drought, heat and salinity) in membranes is electrolyte leakage, which is associated with the variation in the lipidic composition and the amount of these molecules of the cell membrane due to stress conditions. Endophytic fungi are able to induce changes in the lipidic composition of the cell membrane, preventing leakage (Khan et al. 2015; Yan et al. 2019).

Phytohormones have a direct effect on promoting plant growth, and they are also responsible for indirect benefits to plants by modulating the process of adaptation to abiotic stresses. For example, abscisic acid (ABA) is responsible for the closure of stomata, which prevents the excessive loss of water, and changing the expression of genes related to stress responses. The association with endophytic fungi reduces ABA levels (Khan et al. 2015). The phytohormone salicylic acid (SA) directly activates the ASR and regulates the expression of PR proteins, and its interaction with fungal endophytes positively affects SA levels in plants (Khan et al. 2015; Yan et al. 2019).

Khan et al. (2012) tested the inoculation of the endophytic fungus *Paecilomyces formosus* (=*P. maximus*) strain LHL10 in cucumber plants under saline stress. Inoculated plants adapted to salinity conditions showed increase vegetative growth in relation to noninoculated plants. Jan et al. (2019) also described the positive endophytic interaction of *Yarrowia lipolytica*, which mitigated the impact of salinity on maize plants. Inoculated plants were able to improve plant growth attributes, such as the chlorophyll content, electrolyte leakage, leaf relative water, and levels of oxidative enzymes and phytohormones, indicating the possible use of these fungi as biofertilizers under saline conditions. Hamayun et al. (2017) tested the basidiomycetous endophytic fungus *Porostereum spadiceum* AGH786 and assessed its potential to alleviate salt stress and promote the growth of soybean plants by comparing the levels of GA, ABA, and jasmonic acid (JA) in inoculated and control seedlings. Endophytic colonisation was able to maintain high levels of GAs and low levels of ABA and JA, thereby reducing the effect of salinity by modulating phytohormones. The opposite was observed for soybean seedlings presenting a salt-stressed phenotype.

In another study, stress tolerance to high temperature was provided to sunflower and soybean by the endophytic strain *Aspergillus niger* (SonchL-7). Fungal inoculation promoted and increased plant height, biomass and chlorophyll content, in addition to significantly reducing lipid peroxidation and the concentration of ROS during heat stress at 40°C (Ismail et al. 2020). Tolerance to heavy metals is also induced by the interaction of plants with endophytic fungi, and this reaction is similar to that in wheat plants that receive IAA-producing *Penicillium roqueforti* in soil presenting Ni, Cd, Cu, Zn and Pb. The secretion of IAA is responsible for restricting the transfer of heavy metals from soil to plants, and the presence of the fungus improves nutrient uptake and plant growth (Ikram et al. 2018).

In relation to biotic stress, the main defences against pathogens, herbivores and nematodes are the production of secondary metabolites and the activation of systemic resistance by endophytic fungi (Latz et al. 2018; Yan et al. 2019; Poveda et al. 2020). Other possible mechanisms include mycoparasitism and competition. First, one fungus obtains nutrients directly from other fungi, even by causing the death of parasitised cells or obtaining nutrition from living cells (Latz et al. 2018). This kind of interaction is very hard to confirm in endophytic interactions, and it is suggested to be not very important in endophytic action (Card et al. 2016). Competition can occur for space and available nutrients; therefore, endophytic fungi can occupy the niche that could be used by a pathogen if they perform rapid colonisation and scavenging of plant nutrients (Rodriguez et al., 2009; Latz et al. 2018; Yan et al. 2019).

A considerable number of studies in the literature have focused on plant protection from biotic stresses promoted by endophytic colonisation of several fungal species. Many of these studies were performed with *Serependita indica*, which can develop endophytically in different crops, promoting protection against many pathogens (reviewed in Lugtenberg et al. 2016). Other examples, such as Bader et al., 2020, demonstrated the activity of endophytic *T. harzianum* against *Fusarium oxysporum* in tomato plants. Zhou et al. (2018) reported how the endophytic colonisation of cotton plants by *Phialemonium inflatum*, performed via exposure of the seeds to fungal inoculum, was able to suppress the penetration of *Meloidogyne incognita* nematodes into the roots and the formation of galls and affected their reproduction.

Additionally, the effect of endophytic colonisation on plant growth and pest response has been tested. For example, Dash et al. (2018) inoculated *B. bassiana, Isaria* (=*Cordyceps*) *fumosorosea* and *Lecanicillium* (=*Akanthomyces*) *lecanii* in *P. vulgaris* seeds to evaluate the endophytic colonisation ability and plant fitness and its effect on two-spotted spider mite (TSSM), *Tetranychus urticae*. The authors found that all tested strains were able to establish endophytic colonisation of bean plants, and they were recovered from both roots, stems and leaves. Plants whose seeds were treated had positive effects on their development, including plant height and increased fresh biomass of shoots and roots. In addition, mites that fed on plants colonised by fungi showed significantly reduced survival rates, and the negative effect of endophytic colonisation was detected in successive generations of spider mites.

Many other studies in the recent literature can be used as examples of the benefits described above and demonstrate that research has been conducted to evaluate more than one possible benefit that can be provided by fungal endophytic colonisation. *Phoma glomerata* (LWL2) and *Penicilllium* sp. (LWL3) were described as capable of establishing endophytic interactions with cucumber plants, which show significantly increased biomass and better growth under water and salt stress conditions. The symbiotic association increased the assimilation of essential nutrients, such as potassium, calcium and magnesium and reduced the effects of sodium toxicity during saline stress. In addition, modulation of the production of abscisic, jasmonic and salicylic acids was found, proving that the fungi reprogrammed the growth of plants under stress conditions (Waqas et al. 2012).

Lopez and Sword (2015) evaluated the effect of endophytic colonisation of *P. lilacinum* and *B. bassiana* on cotton plants and demonstrated how both fungi were able to increase dry mass and the number of flowers on the plants. Jaber and Enkerli (2017) demonstrated in their study how strains of *B. bassiana* and *M. brunneum* were able to establish endophytic colonisation of *Vicia faba* and promote plant growth of individuals that received treatment via seeds, and they highlighted the importance of the time of exposure of the seed to the fungal inoculum in the colonisation ability and consequent promotion of beneficial effects to the plants. Subsequently, Lr (2018) described how the same species were able to systematically colonise the aerial part and roots of wheat (*T. aestivum*) and promote plant growth (shoot height, root length, and fresh root and shoot weights). Moreover, the study revealed that endophytic colonisation negatively affected the pathogen *Fusarium culmorum*, one of the main causal agents of crown and root rot in wheat.

Krell et al. (2018) were the first to describe the endophytic colonisation of potatoes by *M. brunneum*, and they evaluated the effect of endophytic interactions in deficient and fertilised soil conditions. The fungus was able to positively alter aspects related to growth promotion, especially in treatments where the soil was poor, and under this same condition, endophytic colonisation was intensified. Plants supplied with encapsulated *M. brunneum* presented significantly improved quantum yields of photosystem II, reduced stomatal conductance, enhanced water use efficiency and led to higher biomass, leaf surface development and nitrogen and phosphorus contents.

The benefits of endophytic colonisation were also reported in maize plants in the study of Ahmad et al. (2020), who showed that endophytic colonisation of *M. robertsii* in these plants was able to promote plant growth, increase plant biomass, modulate the expression of defence genes and suppress the development of insect larvae fed to plants that received fungal inoculation. Significant differences in the expression of genes related to the biosynthetic pathways of JA and SA indicate the “priming effect” of the defence system, which guarantees a more effective response in future exposures to possible pathogens and stress conditions. Endophytic colonisation was found in 91% of the plants whose seeds received fungal inoculation, and the high recovery rate of the fungus from shoots and roots indicated the ability of systemic colonisation by *M. robertsii*.

These studies highlighted the potential of several species of fungi to provide benefits to their hosts through endophytic interactions. A considerable number of fungal strains already present widespread use as biocontrol agents (BCAs), especially those belonging to the genera *Metarhizium* and *Beauveria*, as entomopathogens. The deepening of studies involving interactions between microorganisms considered beneficial to plants and their hosts has shown that the plant genome interacts with microorganisms, which has allowed the exploration of a new aspect in the search for more sustainable agriculture (Card et al. 2016).

## 5. Necessary cares about using endophytic fungi in agriculture

It is well-known that some plant growth-promoting endophytes can present some harmful effects on humans and other vertebrates. However, in this context, the use of endophytic fungi is promiser because most surveys involving endophytic microbial toxicity to humans are related to bacterial strains, including species from several genera like *Burkholderia, Enterobacter, Herbaspirillum, Ochrobactrum, Pseudomonas, Ralstonia, Staphylococcus*, and *Stenotrophomonas* (Berg et al. 2005; Mendes et al. 2013). The pathogenic bacteria can reach plant tissues by contaminated manure, irrigation water, seeds, or animals, being able to survive in the soil and colonise plants which indicates the existence of a continuum, even between hosts from different kingdoms (Mendes et al. 2013).

In the case of fungi, negative impacts on vertebrates’ health are registered in studies involving especially *Epichloë* (=*Neotyphodium*) spp. which endophytically colonise grasses. These fungi are responsible for the production and accumulation of alkaloids in plant tissues and these molecules are toxic to several invertebrates and some vertebrates, especially livestock (Faeth 2002; Gimenez et al. 2007; Faeth and Saari 2012). However, only a few studies report the influence of these molecules on vertebrates and it has been assumed that their toxicity to these animals is low and, about agricultural field application of these endophytic fungi, deep research on toxic endophytes by analysing the profile of alkaloids produced by each species, allowed the selection of those which can promote herbivory protection from invertebrates in the field causing no harmful effects on vertebrates (Faeth 2002; Faeth and Saari 2012; Finch et al. 2013). Finch et al. (2013) described the use of ryegrass endophytically colonised with two distinct strains of *Epichloë festucae* (=*Neotyphodium lolii*) to feed dairy cows. The authors detected the presence of the alkaloids produced by the fungus in the milk, however at concentration levels that are considered safe for human consumption. Furthermore, no toxic effects were presented by the animals.

Besides grass endophytes, other endophytic fungi can produce mycotoxins able to harm human health (Chitnis et al. 2020). The fact that some microbes can be noxious for humans and other vertebrates through endophytic colonisation of plants is enough to guarantee special attention to their use as biological products. Therefore, research must be developed to avoid the selection of any known (plant or human) pathogen (Murphy et al. 2018).

## 6. Endophytics as bioproducts: easy, promising and timely

The advantages of using fungi (and other biological agents) in agriculture are already well known and include (i) greater biosafety, (ii) less environmental and human health risk; (iii) specificity with the target pest (without affecting beneficial microorganisms or insects, for example); (iv) efficiency even in small quantities; (v) multiplication (controlled by the plant and the rest of the microbiota); (vi) no recalcitrance, such as chemicals; and (vii) no promotion of the selection of resistant pests; and (viii) use in integrated pest management or in the traditional cultivation system (Berg 2009).

The use of fungi as agents of biological control, especially in the case of entomopathogens, has become widespread as sustainable alternatives to chemical control. The limitation of its use remains mainly in the exposure to UV radiation and low humidity found in the agricultural environment, in addition to problems related to their application in the field (Vega 2018). In this context, as endophytes, fungi can overcome barriers that have traditionally limited their greatest application because they are found inside plants, and they show even greater advantages for those that can be vertically transmitted (which has been rarely explored). Commercially, this finding implies that complex formulations and techniques for application in the field will no longer be needed (Card et al. 2016).

Another recent theme is that disease modification by endophytes is context-dependent, i.e. it depends on biotic and abiotic factors of the environment, host plants and/or pathogens. Variations in pH, temperature and humidity can influence the antagonistic activity of endophytes. Simultaneously, the plant’s own microbiome interacts with the endophyte and may be responsible for variations in its response to the pathogen (Busby et al. 2016). The response to disease severity is also determined by the host plant and the pathogen; that is, in the same plant, the response to different pathogens may not be the same, as in different plants, the response may vary for the same pathogen. In the latter case, the variation in responses is closely related to the defences of the host plant, influencing endophytic colonisation. Thus, inoculation with the same endophyte may result in different effects on the severity of the disease. Unfortunately, few studies specifically indicate context dependency by modifying potential factors and keeping others constant (Busby et al. 2016). The in-depth study of the effect of these factors is essential for the successful evolution of the strategy for using endophytic fungi in the agricultural process.

Such research is even more important considering the commercial context related to biological agents. Globally, information on the use of biological products has become widespread, and with a better understanding of what they consist of and the advantages of their use, in addition to the economic viability of their application, their use has intensified. In 2004, the global biocontrol market was valued at approximately US$ 588 million (Berg 2009), while it is estimated that in this year (2020), it will move US$ 5 billion, with Latin America responsible for the movement of US$ 800 million of this total (Dunham and Trimmer 2020). Brazil is the world leader in the adoption of organic products, and the number of products in the Brazilian market has doubled since 2017, moving more than US 120 USD million in 2019 and with the expectation of an even more promising market in 2020 (MAPA – Ministério da Agricultura, Pecuária e Abastecimento 2020).

## 7. Conclusions and future perspectives

Despite being a technology developed decades ago, the use of fungal species as biological control agents has increased exponentially in recent years. With the growing concern about the mitigation of environmental impacts caused by agricultural processes in nature and in the search for healthier foods free of chemical compounds that are harmful to health, scientific research is developing towards the exploitation of these organisms for this purpose. The ability of endophytic colonisation of crops demonstrated by several fungal species, including those historically used for pest control, has proven to be a very potential mechanism to reach the desired sustainability in agriculture. As stated in this review, endophytic fungi are beneficial because they provide several direct and indirect benefits to crop plants, and it is possible to assume that no synthetic molecule is able to provide such a great range of positive interactions as these microorganisms. Thus, the use of endophytic fungi proves to be an alternative of great potential in the fields of biocontrol, biostimulation and biofertilization, demonstrating that such organisms are a powerful tool for research and enterprises.
